# Prenatal Factors Contribute to the Emergence of Kwashiorkor or Marasmus in Severe Undernutrition: Evidence for the Predictive Adaptation Model

**DOI:** 10.1371/journal.pone.0035907

**Published:** 2012-04-30

**Authors:** Terrence E. Forrester, Asha V. Badaloo, Michael S. Boyne, Clive Osmond, Debbie Thompson, Curtis Green, Carolyn Taylor-Bryan, Alan Barnett, Suzanne Soares-Wynter, Mark A. Hanson, Alan S. Beedle, Peter D. Gluckman

**Affiliations:** 1 Tropical Metabolism Research Unit, Tropical Medicine Research Institute, University of the West Indies, Mona, Kingston, Jamaica; 2 MRC Lifecourse Epidemiology Unit, University of Southampton, Southampton, United Kingdom; 3 Institute of Developmental Sciences, University of Southampton, Southampton, United Kingdom; 4 Liggins Institute and National Research Centre for Growth and Development, The University of Auckland, Auckland, New Zealand; Aga Khan University, Pakistan

## Abstract

**Background:**

Severe acute malnutrition in childhood manifests as oedematous (kwashiorkor, marasmic kwashiorkor) and non-oedematous (marasmus) syndromes with very different prognoses. Kwashiorkor differs from marasmus in the patterns of protein, amino acid and lipid metabolism when patients are acutely ill as well as after rehabilitation to ideal weight for height. Metabolic patterns among marasmic patients define them as metabolically thrifty, while kwashiorkor patients function as metabolically profligate. Such differences might underlie syndromic presentation and prognosis. However, no fundamental explanation exists for these differences in metabolism, nor clinical pictures, given similar exposures to undernutrition. We hypothesized that different developmental trajectories underlie these clinical-metabolic phenotypes: if so this would be strong evidence in support of predictive adaptation model of developmental plasticity.

**Methodology/Principal Findings:**

We reviewed the records of all children admitted with severe acute malnutrition to the Tropical Metabolism Research Unit Ward of the University Hospital of the West Indies, Kingston, Jamaica during 1962–1992. We used Wellcome criteria to establish the diagnoses of kwashiorkor (n = 391), marasmus (n = 383), and marasmic-kwashiorkor (n = 375). We recorded participants' birth weights, as determined from maternal recall at the time of admission. Those who developed kwashiorkor had 333 g (95% confidence interval 217 to 449, p<0.001) higher mean birthweight than those who developed marasmus.

**Conclusions/Significance:**

These data are consistent with a model suggesting that plastic mechanisms operative *in utero* induce potential marasmics to develop with a metabolic physiology more able to adapt to postnatal undernutrition than those of higher birthweight. Given the different mortality risks of these different syndromes, this observation is supportive of the predictive adaptive response hypothesis and is the first empirical demonstration of the advantageous effects of such a response in humans. The study has implications for understanding pathways to obesity and its cardio-metabolic co-morbidities in poor countries and for famine intervention programs.

## Introduction

Each year nine million children under age five years die: malnutrition contributes to one-third of these deaths [1]. Sustained undernutrition in childhood can lead to distinct clinical syndromes of severe acute malnutrition: oedematous (kwashiorkor, marasmic kwashiorkor) and non-oedematous (marasmus). The mortality rate of kwashiorkor is much higher that for marasmus. There is currently no explanation of why some children waste progressively without developing oedema, while others waste less but develop oedema [2]. Patients with kwashiorkor and marasmus differ also in body composition. Thus, when children die of kwashiorkor, they still have significant tissue reserves of protein and fat, as these stores are mobilized inadequately during the disease process [3]. On the other hand, children with marasmus are better able to sustain drawdown from protein and lipid stores. While they have greater tissue wasting on presentation, they have higher survival rates. No differences in pre-morbid dietary intake are reliably found [2].

Kwashiorkor and marasmic patients also display different patterns of intermediary metabolism [4]–[8]. In the acute stage, down regulation of protein turnover is greater in kwashiorkor than in marasmus. Lipid turnover is also lower in kwashiorkor patients. They also have lower intracellular concentrations and synthesis rates of the antioxidant glutathione [3], [4], [8]–[9]. This different metabolic response to severe undernutrition may underlie the ability of marasmic patients to sustain amino acid and lipid supply for intermediary metabolism during the period of decreased dietary intake. In kwashiorkor, however, the greater suppression of protein breakdown creates shortages of essential and conditionally essential amino acids, and these insufficiencies impair protein metabolism, while the reduced availability of lipid impairs energy metabolism. Patients with kwashiorkor are thus likely to suffer metabolic disorganization even while retaining greater tissue stores than in marasmus. Following recovery, protein turnover is approximately 30% faster, and oxidative disposal of lipids is approximately 35% higher, in kwashiorkor survivors than in marasmus [4]–[6].

These fundamental differences in acute and post-recovery metabolism led us to hypothesize that kwashiorkor and marasmus represent differential responses to the same nutritional insult based on pre-existing metabolic differences [4], [10], [11]. Such metabolic differences might arise from exposures *in utero* that induce developmentally plastic responses that match the fetus' metabolism to the anticipated postnatal environment [10], a proposed process labeled predictive adaptive responses [11]. A more limiting prenatal environment will, in addition, reduce fetal growth, reflected in lower birthweight. Such a model is supported by experimental data but has not been directly tested in humans [12]. From such a model we hypothesized that children of lower birthweight would more likely possess a metabolic phenotype which, upon exposure to severe undernutrition, would develop marasmus which has a lower mortaility rate, while those of higher birthweight would develop kwashiorkor, a syndrome less appropriate for conditions of postnatal undernutrition.

## Methods

We reviewed the admission records for all patients who had been admitted to the Tropical Metabolism Research Unit, University of the West Indies, Kingston, Jamaica with severe acute malnutrition between 1963 and 1993. We abstracted clinical (age, gender, extent of oedema), anthropometric (weight and height at admission), and survival data as well as recalled birth weight. Birth weights that were recalled by the mother at the time of admission have been shown to be highly correlated with recorded birthweight [13].

We used the Wellcome criteria to make the diagnosis: weight for age <60% without oedema for marasmus, and weight for age 60–80% with oedema for kwashiorkor. Marasmic-kwashiorkor had weight for age of <60% but had oedema. Children who were 60–80% weight for age without oedema were classified as undernourished.

### Ethics

The study was approved by the Faculty of Medical Sciences Ethics Committee, University of the West Indies.

### Statistical methods

We used linear regression analysis to estimate the difference in birth weight by diagnosis, controlling for sex.

## Results

1,336 patients were admitted to the Tropical Metabolism Research Unit Ward with severe acute malnutrition. Details of their clinical and anthropometric data, survival and birth weights are shown in Table 1 according to diagnosis.

**Table 1:** Clinical, anthropometric, survival and birth weight data of 1336 patients who had been admitted to the Tropical Metabolism Research Unit with severe acute malnutrition between 1963 and 1992.

	Marasmus (n = 383)	Marasmic-Kwashiorkor (n = 375)	Kwashiorkor (n = 391)	Undernourished (n = 187)
**Males (%)**	66.3	68.3	56.0	58.8
**Oedema**	No	Yes	Yes	No
**Died during admission (%)**	1.6	4.6	5.7	1.1

	Mean (SD)	Mean (SD)	Mean (SD)	Mean (SD)
**Age at admission (months)**	11.6 (7.3)	11.9 (7.4)	12.4 (5.9)	14.7 (8.4)
**Height at admission (% expected)**	84.0 (5.9)	84.5 (5.1)	90.6 (4.1)	90.6 (4.1)
**Weight at admission (% expected)**	49.4 (7.4)	51.3 (6.5)	68.4 (5.6)	66.3 (4.8)
**Birth weight of males (g)**	2833 (707)	2906 (644)	3139 (634)	3026 (840)
**Birth weight of females (g)**	2633 (752)	2778 (712)	3004 (682)	2806 (569)

**Figure 1:**
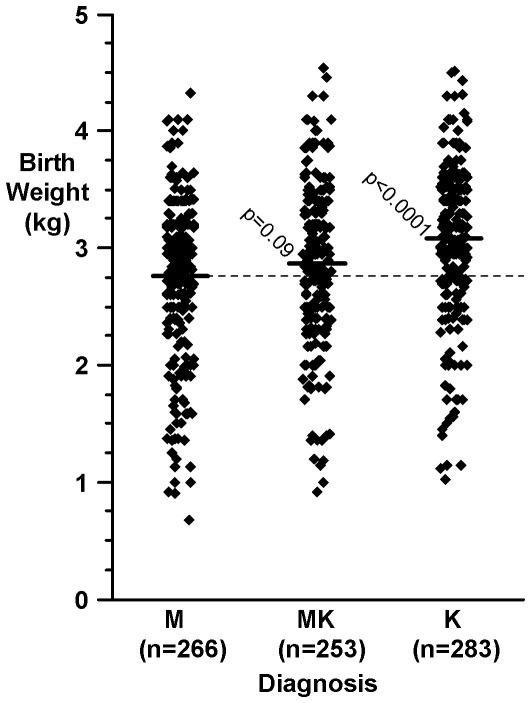
Birth weights of survivors of severe acute malnutrition. Key: M = marasmus; K = kwashiorkor; MK = marasmic kwashiorkor. The horizontal bars are mean values.

Controlling for sex, the mean birth weight of children with kwashiorkor was 333 g (95% confidence interval 217 to 449, p<0.001) more than those with marasmus. The equivalent figures for males and females were 307 (155 to 458) and 371 (188 to 553) respectively. Birth weights for children with marasmic-kwashiorkor were intermediate (Figure 1). Adjusting for age, sex, weight for age (percent) and height for age (percent) those with oedema had birthweights that were 136 g (95% confidence interval 50 to 223, p = 0.002) heavier than those without oedema. To assess the stability of the birthweight difference over time we divided the 1336 admissions into four equal and consecutive groups. The difference in grams between kwashiorkor and marasmic patients over these four periods of the study was, 264, 302, 373, and 419, p<0.02 in all cases

27 males and 20 females died (4.1%). The odds ratio of death was 3.7 (95% confidence interval 1.5 to 9.2, p = 0.005) for kwashiorkor compared to marasmus. Survival was not associated with birth weight; nor did the difference between birth weights of patients with marasmus and kwashiorkor differ according to whether they died during that hospital admission or not.

## Discussion

This study shows that children who experience severe acute malnutrition and develop kwashiorkor, marasmic-kwashiorkor or marasmus have pre-existing developmental differences at birth, as reflected by substantial differences in birth weight. This observation was consistent over the 30 years in the population under study.

This finding provides a developmental explanation for the disparate syndromes of severe malnutrition. It implies that underlying developmental attributes play a role in determining which syndrome is expressed when children are exposed to severe undernutrition. We propose that the developmental environment induces plastic changes affecting the development of metabolic control that can manifest as distinctly different phenotypes under extreme conditions. In the rodent, very different metabolic phenotypes with associated epigenetic changes can be induced by different prenatal nutritional conditions and these offspring respond very differently to postnatal manipulations such as leptin administration [12] or exposure to high fat diets [14]. Persistent epigenetic changes into adulthood have been reported in people exposed to famine *in utero* [15]. The epigenetic state of children in a Western population at birth can be related to mothers' nutritional intake during pregnancy and in turn to later patterns of body compositional development [16].

It has been suggested that developmental plasticity is maintained in mammals to allow the fetus and infant to adjust their development to anticipated future nutritional and other environments. Two classes of developmentally plastic response have been described [17]. The first is immediately required to promote survival of the disadvantaged fetus; growth retardation is such a response. The second class is delayed or predicted responses where the developmental trajectory is altered for predicted advantage to promote survival until puberty and the opportunity to reproduce. The latter class, referred to as a predictive adaptive response, may become disadvantageous later in life if the prediction, based on inadequate transplacental nutrition, is for a low nutritional environment and the offspring faces nutritional excess. However, when the disadvantage presented by such an environment occurs after the reproductive phase, it is likely to be essentially invisible in evolutionary terms. Both classes of response can be induced in the same individual, giving rise to the often reported association between low birthweight and later disease risk. There is considerable experimental support for such a model but its applicability to humans has not been previously directly demonstrated.

The differences in metabolic and clinical attributes between marasmus and kwashiorkor provide a clear demonstration of the pre-pubertal fitness advantage of a predictive adaptive response induced by poor transplacental nutrition, as indicated by lower birthweight. The children born with lower birthweight had a metabolic response to severe undernutrition that aided survival, resulting in the more benign syndrome of marasmus. This finding provides the first direct evidence in humans in support of the fitness-enhancing effects in childhood of anticipatory responses *in utero*.

Nineteen million children worldwide every year become severely wasted [18], [19]. Some of these present with marasmus. If those who develop this syndrome of wasting without oedema possess similar metabolic characteristics as observed in our studies, then we propose that they represent a group with a higher risk of developing obesity and co-morbidities in later childhood, adolescence and adulthood upon exposure to a liberal dietary energy intake [20]. Across the globe such exposure is almost ubiquitous, and exists even in poor countries because of the impacts that globalization and economic transformation have on making food energy more available at an affordable price to almost all their population [21]. On the other hand, individuals with the metabolic architecture seen in kwashiorkor patients are at risk of developing the oedematous syndrome when subjected to severe undernutrition in childhood. Thus, in famine situations, greater attention may need to be paid to children of higher birthweight as they may be at greater risk. We further propose that survivors of kwashiorkor will have a differential and lesser risk of obesity and its cardio-metabolic co-morbid pathologies in adult life.

### Limitations

As this was a retrospective cohort there is a danger of misclassification. However, we sought to reduce this bias by reviewing all admission clinical findings and derived independent diagnoses of kwashiorkor, marasmus and marasmic-kwashiorkor using the Wellcome Classification. Birthweight recall by mothers of the subjects when subjects had been admitted as children might have been inaccurate. However, the validity of such recall has been established in our setting (13). Errors of recall are likely to occur in a way that does not depend on diagnosis, and so are likely to blunt rather than generate birth weight differences between diagnoses. Because the syndromes use weight at admission in their definition, the observed association between syndrome and birth weight may result from a mixture of two effects – an in utero developmental effect and the tracking of weight as children age. However, using the presence of oedema alone as the basis of analysis gives a similar conclusion suggesting that the finding reported here is not due to any confounding effect of birthweight on later weight or height gain.
